# Clinical outcomes of graves’ ophthalmopathy treated with intensity modulated radiation therapy

**DOI:** 10.1186/s13014-017-0908-7

**Published:** 2017-11-06

**Authors:** Yong-Jiang Li, Yong Luo, Wei-Min He, Ping Li, Feng Wang

**Affiliations:** 1 Department of Oncology, West China Hospital, Sichuan University, No. 37 Guoxuexiang, Chengdu, 610041 People’s Republic of China; 2 Deapartment of Ophthalmology, West China Hospital, Sichuan University, Chengdu, People’s Republic of China

**Keywords:** Graves’ ophthalmopathy, IMRT, Clinical outcomes

## Abstract

**Background:**

Radiation for Graves’ ophthalmopathy (GO) has traditionally utilized lateral opposing fields (LOF) or three-dimensional conformal radiotherapy (3DCRT) technique. The current study was conducted to report clinical outcomes and therapeutic effects of intensity modulated radiation therapy (IMRT) in treating GO patients.

**Methods:**

One hundred sixteen patients with GO were treated with IMRT as initial local therapy between July 2010 and August 2013, with a median follow-up of 62 months (range 45–81 months). Radiotherapy dose was 20 Gy in 10 fractions within two to three weeks. The immediate and long-term response to IMRT was evaluated in GO severity score and in each category of symptoms. Acute and long-term complications were recorded to assess its safety.

**Results:**

Symptom severity score significantly fell from the start of treatment to 4- or 6- month post-IMRT (*P* < 0.01). In total, 85 patients (73.3%) experienced improvement of GO symptoms in the first half-year, and only 4 of them (4.7%) suffered recurrence of the GO symptoms during the subsequent follow-ups. Orbital pain, tearing and extraocular muscle dysfunction had the best treatment reaction to IMRT, while proptosis and blurred vision were the most refractory symptoms. Acute complications were slight and self-limited, mainly including intermittent eye redness in 9 patients (7.8%), sideburns hair loss in 19 patients (16.4%), increased milphosis or madarosis in 23 patients (19.8%) and pseudo-progression of GO symptoms in 15 patients (12.9%). For long-term complications, chronic xerophthalmias occurred in 7 patients (6.03%), cataract developed in 2 patients (1.72%), and all were well-managed by medical interventions. Radiation retinopathy and secondary malignancy was not presented in the cohort.

**Conclusion:**

The study demonstrated that IMRT could serve as a viable option in treating GO patients, with a satisfactory symptom control ability, and relatively slight and acceptable post-radiotherapeutic complications.

## Background

Graves’ ophthalmopathy (GO), an autoimmune orbital inflammatory pathology, is the most common extrathyroidal manifestation of Graves’ disease. GO originates from dysfunction of lymphocytes, autoantibodies and cytokines that lead to edema of the extraocular muscles (EOM) and fats, increasing the volume within bony confine of the orbital cone, and leading to symptoms including orbital pain, proptosis, diplopia, optic nerve compression, exposure keratitis, corneal ulceration and visual damage [1–3]. The goal of GO treatment include relieving ocular pain, reducing diplopia, preserving vision and improving cosmetic appearance. For patients whose GO symptoms are not relieved by normalization of thyroid function, glucocorticoid agents have been traditionally used as the first-line treatment method. However, the therapeutic effects of corticoids varied. Approximately 65% of these patients could have their symptoms improved, but relapse is common when the corticoids are reduced or withdrawn [4, 5]. Besides, multiple side-effects including immune system compromise, hyperglycemia and hypertension could restrict the usage of corticoids administration.

Retro-orbital radiotherapy is useful in treating GO patients who are not sensitive to or cannot tolerated corticoids, or those with recurrent symptoms after corticoids therapy. The mechanism is mainly through the non-specific anti-inflammatory effects of radiotherapy, suppressing radiosensitive infiltrating lymphocytes, inhibiting fibroblast proliferation and mucopolysaccharide secretion [6]. Although the radiotherapy is slower to reveal therapeutic effects than corticoids, it could provide a more prolonged protection period [7].

Traditionally, lateral opposing fields (LOF) technique was used for retro-orbital radiotherapy due to its simple and prompt set-up procedure. Nevertheless, LOF has obvious drawbacks of inhomogeneous dose distribution within the target and inadequate dose to the anterior part of ocular structures including EOM insertions and anterior sectional retro-orbital fats which is limited by protection for the lenses. Afterwards, three-dimensional conformal radiotherapy (3DCRT) was introduced in retro-orbital radiotherapy, which could provide better target coverage and superior dose sparing to normal structures, thus better suiting for the retro-orbital radiation.

However, as demonstrated by Lee et al. in the dosimetric study including 10 patients with GO treated with intensity modulated radiation therapy (IMRT) [8], IMRT could provide a much better and more conformal coverage of the targets than 3DCRT in the retro-orbital radiotherapy, generating significantly superior conformity index and homogeneity index. As a result, IMRT could be theoretically more efficient in treating GO, possibly reaching a higher control rate while providing a better preservation to adjacent normal structures including lenses, globes and optical nerves. Nevertheless, as the study is a dosimetric study, no treatment outcome was reported, and the number of GO patients underwent IMRT was limited. Besides, to our knowledge, no study has been done concerning whether the technical and dosimetric superiority of IMRT could translate into clinical benefits in treating GO patients. Therefore, we conduct the current study to report the clinical outcomes and evaluate the therapeutic effects of IMRT in treating GO patients.

## Methods

Our research is in accordance with the Helsinki Declaration and approved by the medical ethics committee of West China Hospital, Sichuan University. Informed consent was obtained from all individual participant included in the study.

The study is a retrospective study based on prospective collection of treatment data. The data of initial disease severity, treatment response, and acute and long-term complications were prospectively collected in clinic when we treated GO patients enrolled in our hospital with retro-orbital IMRT from July 2010 to August 2013. The other data including the basic characteristics were retrospectively collected from the medical records of these patients. Patients were included in the study if the following criteria were met: (1) patients with clinically diagnosed GO; (2) treated with radiotherapy using IMRT technique at our hospital; (3) with complete medical records and laboratory reports; (4) with regular follow-ups and the corresponding clinical data. The patients in the following conditions were excluded: (1) lost to follow-up or died because of other diseases; (2) only patients with hyperthyroidism were included for a more homogenous background, thus patients with hypothyroidism were excluded; (3) had received radiotherapy for GO or decompressive surgery before; (4) treated with other radiotherapy techniques such as LOF or 3DCRT; (5) patients with IMRT treatment for a single eye were also excluded to achieve a homogenous background.

Clinical data of interest were extracted independently by two authors. A Microsoft Excel sheet was designed to collect the following records: (1) patients’ basic characteristics including age, sex and treatment methods for the hyperthyroidism; (2) smoking status (current smoker, former smoker or non-smoker); (3) duration of GO symptoms, previous treatment methods for GO, and disease severity of GO at inclusion; and (4) response to IMRT, and acute and long-term complications.

GO symptom severity was evaluated by utilizing GO symptom scoring system [9], which is based on 8 symptom categories, 5 of which were from the NOSPECS classification system: soft tissue involvement (S), proptosis (P), extraocular muscle involvement (E), corneal involvement (C), sight loss (S), diplopia, orbital pain, and tearing. Each category except tearing was assigned a score of 0, 1 and 2 representing no symptom, mild to moderate symptom, and severe symptom, respectively. For tearing, a score of 0 (no tearing) or 1 (tearing) was assigned. Total GO severity score was the cumulative score of all the listed categories and ranged from 0 to 15. The response to radiotherapy was calculated as [(baseline score – 6 month post-radiotherapy score)/baseline score], and categorized as follows: < 0% - progression, 0% to 10% - no response (NR), 10% to 33% - mild response, 33% to 66% - moderate response, and > 66% - significant response. For individual symptom category, the response was categorized as complete (CR) if the symptom was completely resolved, or partial (PR) if the symptom was improved but not completely resolved.

**Fig. 1:**
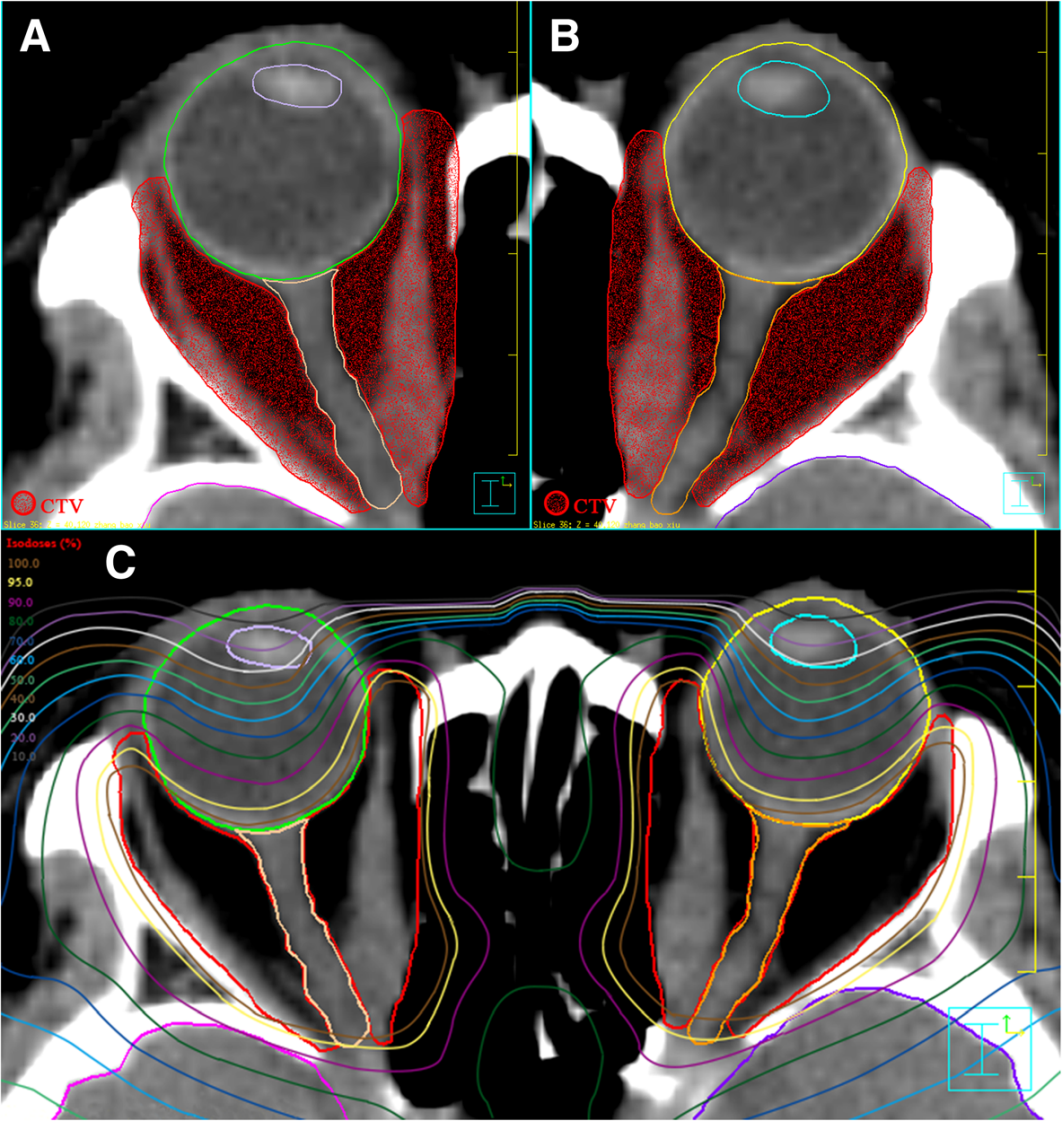
Target volume delineation (a, b) and planned dose distributions (c) of IMRT in treating Grave’s ophthalmopathy

All patients were treated with retro-orbital irradiation using linear accelerator based IMRT technique. Patients were immobilized with a custom-made thermoplastic case, and then computed tomography (CT) scan with slice thickness of 2.5 mm without contrast was performed for image acquisition and target contouring. The clinical target volume (CTV) encompassed the origins to insertions of the extra-ocular muscles and the retro-orbital fatty spaces with the main bulk (Fig. 1A,B). The lenses, globes, optic nerves and lacrimal glands were zoned as organs-at-risk (OAR). A 2 mm concentric margin around the CTV was generated as the planning target volume (PTV). A total dose of 20 Gy was given to each patient in 10 fractions within two to three weeks by reversely planned 7-filed IMRT. All the IMRT plans were verified to ensure that the 90% isodose line cover the PTV before 6MV beam was generated by linear accelerator. The planned dose distribution was shown in the Fig. 1C. After radiotherapy, the patients were followed-up every 2 months for the first half-year, then every 3 months for the first year and every 6 months thereafter unless a specific clinical event emerged.

The statistical analyses were conducted using SPSS Statistics version 22.0 (IBM Corp., Chicago). Descriptive statistics were performed to characterize the patient population, treatment outcomes and complications. The symptom severity scores at different treatment periods were compared by Kruskal-Wallis test and Mann-Whitney U test, and illustrated in Box plots. Chi-squared test and Fisher’s exact test were conducted to identify the predictive factors for significant and moderate response to IMRT. All the statistical analyses were considered significant at two-tailed *P* < 0.05.

## Results

### Patients’ characteristics

The clinicopathological characteristics of these patients are presented in Table 1. Initially, a total of 133 patients were identified at inclusion, and 17 patients were excluded: 7 patients lost to follow-up with incomplete medical records that we could not evaluate their disease development after IMRT, 4 patients had single-eye irradiation, 4 patients have received decompression surgery before radiotherapy, and 2 patients died of other diseases (heart disease and car accident) and their follow-up data were not complete. Finally, a total of 116 patients were enrolled in the current study, including 52 male patients and 64 female patients. The median age was 53 years old (range 23 to 78 years), and the median follow-up was 62 months (range 45 to 81 months). Majority of the patients (*n* = 63, 54.3%) were non-smokers, while 42 patients (36.2%) were current smokers at enrollment and 11 patients (9.5%) were former smokers. The duration of GO prior to radiotherapy was less than 6 months in 41 patients (35.3%), 6 to 18 months in 48 patients (41.4%) and longer than 18 months in 27 patients (23.3%). The disease severity score at enrollment was 2 to 5 in 32 patients (27.6%), 6 to 10 in 63 patients (54.3%) and 11 to 14 in 21 patients (18.1%).

**Table 1:** Basic characteristics of patients

Variables	No.	Percent
Gender		
Female	64	55.2%
Male	52	44.8%
Previous thyroid treatment		
Medication	84	72.4%
Thyroidectomy	14	12.1%
RAI	43	37.1%
None	20	17.2
Smoking status		
Current smoker	42	36.2%
Former smoker	11	9.5%
Non-smoker	63	54.3%
Duration of GO prior to RT		
≤ 6 months	41	35.3%
6–18 months	48	41.4%
> 18 months	27	23.3%
Previous steroid use		
Yes	39	33.6%
No	77	66.4%
Response to previous steroids		
None	10	25.6%
Mild to moderate	29	74.4%
Significant	0	0%
Concurrent steroid use during RT		
Yes	21	18.1%
No	95	81.9%
Symptom severity scores at enrollment		
2 to 5	32	27.6%
6 to 10	63	54.3%
11 to 14	21	18.1%

*RAI* radioactive iodine, *GO* Graves’ ophthalmopathy, *RT* Radiation therapy

### Immediate response

**Fig. 2:**
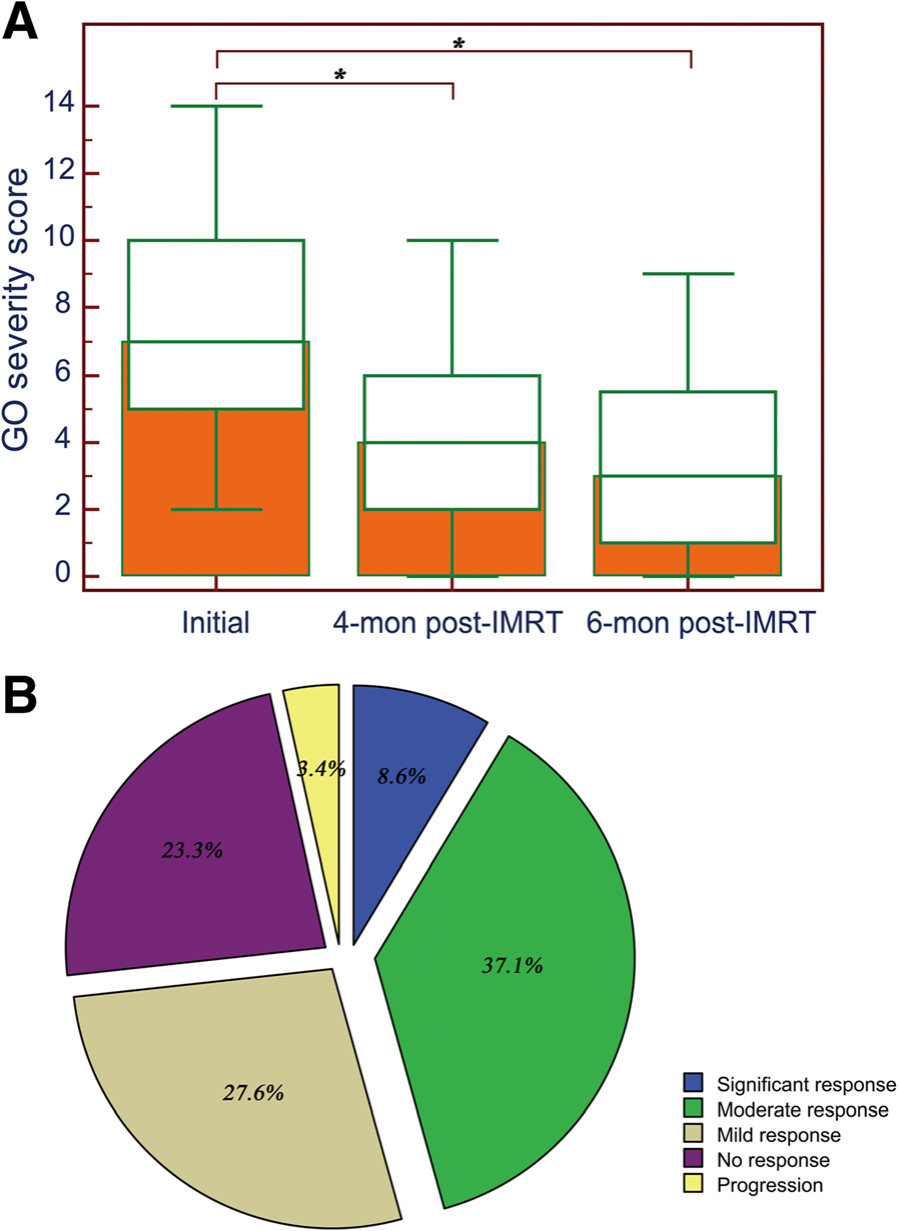
Treatment response to IMRT in the initial 6 months. a The changes in GO symptom severity score; b The percentage of patients with different response degrees

The immediate response of GO symptoms to IMRT has been shown in Fig. 2. The median severity scores at the enrollment, 4 months and 6 months post-IMRT were 7 (range 2–14), 4 (range 0–10) and 3 (range 0–9), respectively. The scores at 4 months (*P* < 0.001) and 6 months (*P* < 0.001) post-IMRT were significantly lower than the initial score (Fig. 2a). For the patients, majority of them (73.3%) experienced improvements of GO symptoms, and more specifically, the overall therapeutic reaction was mild response in 32 patients (27.6%), moderate response in 43 patients (37.1%) and significant response in 10 patients (8.6%). However, 27 patients (23.3%) showed no response to IMRT and 4 patients (3.4%) experienced progression of the GO symptoms (Fig. 2b).

The response to IMRT in the category of individual symptoms at 6 months after irradiation was shown in Table 2. The orbital pain has the best treatment reaction to IMRT, the tearing and EOM dysfunction also had favorable treatment response. In contrast, proptosis and blurred vision were most refractory to IMRT, with CR rates of only 10.3% and 17.4%, respectively.

**Table 2:** Response to IMRT by category of symptoms

Category	No. (%)	CR No.	Percentage	CR & PR No.	Percentage
NOSPECS classification system					
Soft tissue involvement (S)	85 (73.3)	14	16.5%	52	61.2%
Proptosis (P)	78 (67.2)	8	10.3%	32	41.0%
EOM dysfunction (E)	62 (53.4)	20	32.3%	34	54.8%
Corneal involvement (C)	7 (6.0)	2	28.6%	3	42.9%
Sight loss (S)	23 (19.8)	4	17.4%	10	43.5%
Three additional symptom categories					
Orbital pain	45 (38.8)	32	71.1%	37	82.2%
Tearing	50 (43.1)	17	34.0%	38	76.0%
Diplopia	39 (33.6)	9	23.1%	22	56.4%

*PR* Partial response, *CR* complete response, *EOM* extra-ocular muscle

In terms of acute complications, a total of 9 patients (7.8%) developed 1 to 6 times of unilateral or bilateral eye redness, within the periods from start of IMRT to 3 months post-IMRT. Each time, the eye redness could be spontaneously relieved or eliminated within a week. Besides, 19 patients (16.4%) presented slight hair loss at the site around sideburns, and 23 patients (19.8%) complained about the increased milphosis or madarosis, from 1 month to 4 months post-IMRT. Also, the symptoms could be in remission spontaneously after 6 months post-IMRT. In addition, 15 patients (12.9%) reported increased orbital pain accompanied with self-consciously progressed proptosis after the radiotherapy, but the symptoms achieved PR or CR at the 6 months’ follow-up, and this may be possibly caused by the temporary edema of the retro-orbital tissues after radiotherapy.

### Long-term response

The symptom severity score at the last follow-up were not significantly different from the score at 6-month post-IMRT, and had the same median value of 3 points. For the 85 patients with immediate treatment reactions of significant response, moderate response and mild response, only 4 patients (4.7%) suffered obvious exacerbation or recurrence of the GO symptoms, 3 of whom were current smokers and refused to quit smoking. The symptom was maintained or continually improved in the rest patients (*n* = 81, 95.3%). For the 31 patients with no response or progression of GO symptom at 6-month post-IMRT, no obvious changes in the symptom severity occurred during the subsequent follow-ups, and decompression surgery was recommended to those meeting the operation indication.

As for complications, no patients presented with the intermittent eye redness, sideburns hair loss and increased milphosis or madarosis at the last follow-up. Chronic xerophthalmias occurred in 7 patients (6.03%), and were well controlled with administration of artificial tears. Cataracts developed in 2 patients (1.72%), and were dealt successfully with cataract removal and lens replacement. Radiation retinopathy was not presented in the cohort, and no secondary malignancy has been detected.

#### Predictive factors for significant and moderate response to retro-orbital IMRT

The predictive factors for moderate to significant response to retro-orbital IMRT were shown in Table 3. Current smoking status was found to be significantly associated with the moderate to significant treatment response to IMRT (OR 0.40, 95% CI: 0.18–0.89; *P* = 0.025). Factors including age, sex, concurrent steroid use and former smoker were not found to be significantly correlated with the treatment response.

**Table 3:** Predictive factors for significant and moderate response to retro-orbital IMRT

Characteristics	Odds ratio	95% CI	*p*-value
Age (≤ 53 vs. > 53)	1.46	0.68–3.14	0.332
Sex (Male vs. Female)	0.53	0.24–1.16	0.114
Concurrent steroid use during RT (Yes vs. No)	1.83	0.69–4.86	0.227
Smoking status			
Non-smoker	reference		
Former smoker	0.46	0.12–1.72	0.247
Current smoker	0.40	0.18–0.89	0.025

## Discussion

To our knowledge, the current study based on 116 patients firstly provide a comprehensive evaluation on the treatment efficacy and clinical outcomes of IMRT in treating GO. Our findings demonstrated that IMRT is a viable option for GO patients, with a satisfactory symptom control ability and acceptable post-radiotherapeutic complications, and could be recommended to GO patients in clinic.

Historically, LOF technique was utilized in treating GO because of its easy set-up and swift delivery procedures. However, to minimize the dose to the lenses, the beams are either blocked to the anterior portion of the globes or tilted five degrees posteriorly, which would result in the inadequate dose to the EOS insertions and the anterior portion of the retro-orbital fat that are commonly involved in GO. Besides, the distribution within the target is hard to reach homogeneous by LOF technique. As treatment technology developed, 3DCRT gradually replaced two-dimensional radiation therapy in clinic, for the superior target coverage ability and better radiation sparing to the normal structures, which is particularly important in the precise irradiation of the ophthalmic structures.

Afterwards, IMRT emerged as an evolutionary form of 3DCRT, which is able to deliver a dose distribution around a more irregular and complex target volume [10]. Besides, IMRT could achieve steeper dose gradients between tumor targets and normal structures, and thus, reduce the dose to surrounding tissues without reducing planning target volume coverage [11, 12]. As a result, IMRT may be more applicable than 3DCRT in treating GO because of the quite irregular target volume of the retro-ocular structures. The superiority was theoretically demonstrated by Lee et al. in his dosimetric study involving 10 GO patients underwent IMRT [8]. Significantly superior conformity index and homogeneity index were observed in IMRT compared with 3DCRT. In addition, IMRT provided better dose sparing to globes, lenses and optic nerves. However, no treatment results were reported as it is a dosimetric study, and no other studies has been done to provide relevant clinical outcomes. Therefore, the current study was conducted to provide clinical evidences and comprehensively evaluate the therapeutic outcomes of IMRT in treating GO patients.

All our patients received 20Gy to both eyes in 10 fractions over 2 weeks, which is the standard radiotherapy protocol in treating GO. A higher cumulative dose was not reported to provide further benefit, and some studies found that a lower dose was equally effective to 20Gy, but inconsistent results were reported [4, 13, 14]. A randomized study revealed that a radiation protocol with 20 Gy fractioned by 1Gy/week over 20 weeks was more effective and better tolerated than the standard protocol, and even dose of 10 Gy (1 Gy weekly delivered in 10 weeks) could achieve considerable treatment results [13]. Nevertheless, the much prolonged treatment duration made it less practical in radiation department of our hospital. Based on the background, we finally utilized the standard protocol in the current study, as it is most commonly used and of less controversy. However, it should be noted that the aforementioned protocols were established based on the traditional LOF or 3DCRT radiation technology. As IMRT could achieve a better dose differentiation between tumor targets and normal tissues, the standard protocol may not be the most optimized protocol, and it needs to be further clarified in future studies.

We utilized the symptom scoring system to evaluate the GO symptom severity and the treatment response [9]. Initially, according to NOSPECS classification system, 5 general categories were introduced to assess GO signs and symptoms: soft tissue involvement, proptosis, EOS dysfunction, corneal involvement and sight loss [15]. However, due to the known limitations of the NOSPECS system, additional symptom categories of diplopia, orbital pain and tearing were also included to achieve a more comprehensive assessment [15]. In our patients, IMRT exhibited obvious curative effects in relieving GO symptoms. The total severity score significantly fell from the initiation of treatment to 4 months and 6 months post-radiotherapy. Although the difference between scores of 4-month and 6-month post-radiotherapy did not reach statistically significant, a decreasing trend could still be observed. The proportion of patients who had mild to significant improvement of GO symptoms was 73.2%, which is at a relatively high level among the previous studies [16–18]. The response to radiotherapy in the category of individual symptoms exhibited heterogeneity. Proptosis and blurred vision were most responsive to IMRT while orbital pain and tearing was the most refractory symptoms. The response variation suggested that clinicians should take the constellation of symptoms into consideration while selecting GO patients for radiotherapy, and those with proptosis and blurred vision should be aware that the may not have satisfactory benefits from the radiotherapy.

Overall, IMRT was well tolerated in the cohort as no patients required treatment breaks for acute toxicity. The intermittent eye redness, sideburns hair loss and increased milphosis or madarosis were the most common acute complications we recorded, and the symptoms were generally slight and temporary. The relative low doses in treating GO and the high accuracy of IMRT technique could explain the mild acute toxicity. It needs to note that 15 patients (12.9%) in the cohort experienced self-conscious aggravation of the GO symptoms including increased orbital pain and progressed proptosis soon after the radiotherapy, but the symptoms disappeared afterwards, which we considered as the pseudo-progression of GO symptoms during IMRT. The pseudo-progression often occurred in patients with higher levels of initial symptom severity score, and it is possibly caused by the temporary edema of retro-orbital tissues after radiotherapy.

As for the long-term complications, our study exhibited satisfactory safety of IMRT in treating GO patients. The main drawback of IMRT is that dose in painting area is larger than conventional LOF irradiation. During retro-orbital irradiation, lens and lacrimal glands would be affected by the radiation rays. The toxicities of lens and lacrimal glands mainly presented as chronic complications in the long-term, and the patients may develop chronic xerophthalmias and cataract after the radiation treatment. In our cohort, 7 patients developed chronic xerophthalmias and 2 patients developed cataract. Taking into consideration of the fact that the advancing age was also associated with a risk of cataract development, the possibility of cataract formation directly caused by retro-orbital IMRT in clinic practice was less than two cases out of the whole 116 patients. The low radiation doses in treating GO is one reason. According to previous data from patients with orbital lymphoma, a lens opacity may form after a single fraction of 200 cGy [16, 19]. For patients with lens dose of 1500 cGy fractioned during a treatment period, the probability of cataract formation was 12% at 5 years [16]. Secondly, the superiority that IMRT could provide a better preservation to adjacent normal structures may also contribute to the low rate of cataract formation. In our patients, with appropriate field volumes designed to protect the lens, the lens dose was estimated to be under 700 cGy, which was fractionated in 10 fractions over 2 to 3 weeks. As a result, the risk of cataract formation after IMRT for GO treatment is supposed to be relatively small.

Because cataract is easily to be corrected with eye operation, some other possible complications are clinically more severe than cataract. Radiation retinopathy is a sight-limiting complication after ophthalmic radiotherapy, which is characterized by vascular closure, vascular incompetence and resultant loss of vision [20]. The risk of radiation retinopathy is tightly related to total dose, radiation dose rate, the use of radiation sensitizers (e.g. chemotherapeutic agents), and the presence of synchronous systematic disease (e.g. diabetes). According to previous data, the radiation dose that can cause up to 5% radiation retinopathy within 5 years was estimated to be 45 Gy [19, 21]. In our patients, the radiation dose is much less than 45 Gy, and no chemotherapy is needed in treating GO. Thus, it is reasonable to find that no patients in the cohort suffered from the radiation retinopathy. Secondary malignancy is another major concern, but the excess lifetime risk of radiation induced fatal cancer after radiotherapy for GO was calculated to be 7 cases per 1000 persons (0.7%) based on the traditional LOF technique [22]. As the IMRT could significantly reduce the dose sparing in normal tissues, the theoretical risk was felt to be acceptable for the patient population, and that no secondary cancer was detected in out cohort could further verify the view.

Our cohort consisted a relevant high proportion of male patients, which was due to that the population distribution of our cohort had its particularity: Chinese GO patients who were willing to receive IMRT treatment. Because it is a radiotherapy treatment method and has a relevant higher price, there is a trend towards more serious disease status in the cohort. According to a study concerning the clinical characteristics of moderate-to-severe GO in Chinese patients which included 354 patients [23], 52.26% were female patients, which was similar to our study. The underline reasons may be due to that, firstly, the female-to-male ratio decreases correspondingly with the severity of the disease [24], and secondly, the predominance of females over males in the incidence of GO was considerably less in Asian patients [25]. Thirdly, in China, female patients tend to choose primary and second level hospitals for medical service at the beginning of the illness, while male patients always wait and endure until the disease progressed to an unbearable condition [23], and they always concentrate in tertiary hospitals like our center. Thus, it was rational to found that our cohorts consists a relatively high proportion of male patients.

It is needed to mention the general indications and contraindications that we used in clinic in treating these GO patients. The most important indication is that the patients were aware and approval of using radiation therapy method to treat GO, as well as its potential risks including contract formation, radiation retinopathy and secondary malignancy. In addition, the patients should have good general condition without other serious acute diseases. For the contraindications, patients with diabetic eye diseases, or diabetic patients without good glucose control should not be treated with radiation therapy because of the possibly increased risk of retinopathy. In addition, patients with local infections in the radiation area should wait until the infectious area is recovered.

One limitation of the current study is that it is a single-center study. Although we included a relevant large sample among studies concerning GO patients, the results should still be treated with caution. Secondly, although the NOSPECS classification system were incorporated in the study, the CAS system could not be evaluated in these patients due to the insufficient records. The CAS system is needed to be utilized in future studies to achieve a more accurate conclusion.

## Conclusion

In conclusion, the study demonstrated that IMRT is a viable option in treating GO patients, with a satisfactory symptom control ability, and relatively slight and acceptable post-radiotherapeutic complications. The majority of the patients achieved stabilization or improvement of the GO symptoms, and current smoking status was correlated with decreased possibility of favorable symptom response. Cataract formation and chronic xerophthalmias were found as long-term complications and could be well-managed. Radiation retinopathy and secondary malignancy were not presented in the cohort. A total dose of 20 Gy in 10 fractions were utilized in the current study, but the most optimized protocol of IMRT in treating GO patients is still needed to be identified in future studies.

## Abbreviations

3DCRT: Three-dimensional conformal radiotherapy
CR: Complete response
CTV: Clinical target volume
EOM: Extraocular muscles
GO: Graves’ ophthalmopathy
IMRT: Intensity modulated radiation therapy
LOF: Lateral opposing fields
NR: No response
OAR: Organs-at-risk
PR: Partial response
PTV: Planning target volume
